# Choice, quality and patients’ experience: evidence from a Finnish physiotherapy service

**DOI:** 10.1007/s10754-020-09293-z

**Published:** 2021-01-19

**Authors:** Visa Pitkänen, Ismo Linnosmaa

**Affiliations:** 1 Research Department, Social Insurance Institution of Finland, P.O. Box 450, 00056 Helsinki, Finland; 2 Department of Health and Social Management, University of Eastern Finland, P.O. Box 1627, 70211 Kuopio, Finland; 3 Centre for Health and Social Economics, National Institute for Health and Welfare, P.O. Box 30, 00271 Helsinki, Finland

**Keywords:** Choice, Quality, Competition, Demand, Health care, C25, D12, I11

## Abstract

We study the relationship between patient choices and provider quality in a rehabilitation service for disabled patients who receive the service frequently but do not have access to quality information. Previous research has found a positive relationship between patient choices and provider quality in health services that patients typically do not have previous experience or use frequently. We contribute by examining choices of new patients and experienced patients who were either forced to switch or actively switched their provider. In the analysis, we combine register data on patients’ choices and switches with provider quality data from a competitive bidding, and estimate conditional logit choice models. The results show that all patients prefer high-quality providers within short distances. We find that the willingness to travel for quality is highest among new patients and active switchers. These results suggest that new patients and active switchers compare different alternatives more thoroughly, whereas forced switchers choose their new provider in limited time leading into poorer choices.

## Introduction

Patient choice policies have been implemented in many health care services with an aim to promote quality, responsiveness and efficiency (Brekke et al. 2014; Propper et al. 2006). A necessary condition for successful competition is that patients are sensitive to quality differences among providers (Varkevisser et al. 2012). However, it is not self-evident that demand increases with quality in health care services, where choices are often made under asymmetric information (Arrow 1963). Even when patients are well informed, they face costs in switching providers (Gravelle and Masiero 2000). Patients also face a trade-off between quality and travel costs (Beukers et al. 2014) and they usually bypass the nearest provider only for a particular reason (Varkevisser and Van der Geest 2007).

The empirical literature on patient choice has grown in recent years because of widespread choice policies and the availability of quality indicators regarding health care providers. Previous studies have usually found a positive relationship between patient demand and quality in primary and specialized health care services (Beckert et al. 2012; Gaynor et al. 2016; Gutacker et al. 2016; Moscelli et al. 2016; Santos et al. 2017; Smith et al. 2018; Varkevisser et al. 2012). However, the positive relationship has also been found in services where no public information is available, perhaps due to previous experiences shared within patient networks (Gutacker et al. 2016; Moscone et al. 2012).

In this paper, we examine the relationship between patient choices and provider quality in a health service that is provided to patients on a weekly basis for several years. Patients can choose from a very large set of alternative providers but they do not have access to comparable quality information regarding the providers. The providers are selected every four years in a competitive bidding based on their price and quality. Our data allows us to examine the choices of patients choosing a provider for the first time (henceforth, *new patients*), as well as choices of experienced patients who either initiated the switch (*active switchers*) or were forced to switch their provider (*forced switchers*) because their initial provider was no longer available in the pool of providers.

There are three theoretical aspects related to patient choice and provider quality in health services where patients receive the service frequently but do not have access to quality information. First, experienced patients may learn about certain quality aspects when they receive the health service frequently for many years (Biørn and Godager 2010). Second, even if providers’ quality is not observable to patients prior to their first visit, providers have strong incentives to produce high quality to maintain their reputation with their usual patients (Kranton 2003). Third, providers have an incentive to give signals about their true quality, in an attempt to reduce errors made by patients (Gravelle and Masiero 2000).

Previous literature has studied the choice of hospital in treatments such as hip replacement (Gutacker et al. 2016) or angioplasty (Varkevisser et al. 2012), treatments from which the patients do not usually have any previous experience. We are the first to analyze differences between the choices of new and experienced patients, who are presumably, informed differently about the quality and characteristics of the service and alternative providers. We also study the difference between patients who travel and those who receive the service at their home. We estimate conditional logit choice models using register data on choices in the 2011–2014 contract period.

We find that, in general, all patients prefer high-quality providers within short distances. The results show that the willingness to travel for quality is highest among new patients and active switchers. These results suggest that new patients search for very experienced providers, active switchers have learned about certain quality aspects and forced switchers might have had only limited time to choose their new provider. We also find that patients who travel prefer shorter distances and high-quality facilities compared to patients who receive the treatment at home.

## Institutional setting

The institutional context of our study is an intensive medical rehabilitation service financed by the Social Insurance Institution of Finland (Kela), which is the largest single financer of rehabilitation services in Finland. The focus of our study is the individual outpatient physiotherapy service, which is the most common form of medical rehabilitation. The physiotherapy was organized for 14,756 patients and the costs were 73.5 million euros in 2015 (SVT 2016). Kela is obliged by law to arrange the service for severely disabled persons under 65 years of age who face problems managing daily activities and fulfil the eligibility criteria defined in the Rehabilitation Act (566/2005). The physiotherapy is based on a written rehabilitation plan drawn up with a physician for one to three years at a time. Patients typically receive the physiotherapy in sessions of 45 or 60 min, once or twice a week, for several years. The aim is to promote the patients’ autonomy and improve or maintain their work capacity and functioning. The service is provided free of charge for all patients.

Kela’s insurance districts are responsible for organizing the service for the local population. The districts acquire the service from private physiotherapy providers using a competitive bidding every 4 years. Providers give information on their quality, set a price for a 45-min session and report their annual capacity when they participate in the procurement. This study concentrates of patients’ behavior in the 2011–2014 contract period, for which the procurement was organized in 2010. Each insurance district organized the procurement in a predefined and similar manner.^1^ Providers that fulfilled the minimum requirements were ranked based on their quality-price ratio, and the districts offered a contract to a number of providers based on local demand. Providers that signed the contract formed the pool, from which the patients were able to choose. Providers send their invoice directly to Kela, which pays for each visit based on each provider’s accepted price. After the 2010 procurement, the pool included a total of 1297 providers.

In the 2010 procurement, the districts evaluated providers in six quality categories that were education, experience, facilities, equipment, language skills and Kela’s quality standard. The overall maximum score for quality was 105.^2^ In practice, the providers filled procurement forms that included questions about the quality aspects. Providers were also required to attach certified copies of their physiotherapists’ education. Even though Kela audits some providers occasionally, it did not monitor contracted providers’ reported quality during the contract period. However, all providers adhered to maintain their quality at the level of their tender for the entire contract period.

Kela has emphasized the free choice of providers in all of its rehabilitation services since 2011.^3^ The aim has been to promote competition and involve individuals in decisions related to their rehabilitation. Patients are free to choose from all accepted providers. Although the districts collect quality information in the competitive bidding, this information is not available to patients. Also, Kela officials can neither recommend nor favor any provider. Thus, patients may have problems finding information about high-quality providers. However, Kela’s local offices have provided lists of alternative providers in the district and in 2011 Kela also established a website^4^ that lists the providers in a municipality or district. Thus, patients are likely to be aware of alternative providers in their area. They also gather information from many formal and informal sources, such as health care professionals, Kela officials, peer groups and provider websites^5^ (Pitkänen and Pekola 2016).

Patients can switch providers at any time during their rehabilitation process. However, switching always results in some costs. In this service, patients have to inform the local insurance district about their new provider. Switching is also associated with the discontinuation of established relationships especially when patients have repeated visits with the same provider (Anell et al. 2017). Most physiotherapy patients do indeed commit themselves to one provider for a long time. The switching behavior in this service can be divided into two different categories: first, patients can initiate the switch of providers. However, previous literature has shown that, usually, patients do not actively search for information nor switch providers (Victoor et al. 2012). Second, patients have been forced to switch their provider when their previous provider was no longer included in the pool of providers or ended their contract during the contract period.^6^ Often the forced switch comes as a surprise and patients need to make a new choice in relatively short time period. The previous provider is often involved in the choice of the new provider and they are also required to inform the new provider about the patient’s rehabilitation needs and process. Thus, forced switchers can be considered as a random sample of experienced patients who had to choose a new provider, whereas other switchers are most likely a selective group of the most active experienced patients.

Patients receive the physiotherapy either at home or at the provider’s facilities.^7^ Patients can apply to receive the service at their home if their capability to travel is limited and their home is suitable for the physiotherapy sessions. If the service is received at home, patients are indifferent about travel costs and time. Kela also pays providers extra based on the length of their travel and the given session.^8^ Patients that travel to the provider’s facilities also get reimbursements from Kela for their travel costs. The reimbursement is calculated based on travel costs to the nearest alternative provider, and these costs have an annual deductible.^9^ Therefore, it is likely that patients who travel prefer shorter distances than patients who receive the service at home.

## Data

We have constructed our data by linking patient-level register data on rehabilitation applications and invoices from the years 2010–2014 with provider quality data that was collected in the 2010 competitive bidding. In our analysis we focus on choices in the 2011–2014 contract period for four main reasons. First, free choice of provider has been emphasized in the service since 2011. Second, the eligibility criteria for the service and the insurance districts remained the same during the contract period. Third, providers adhered to maintain their quality at the same level for this entire period and therefore choices across these years should be comparable. Fourth, Kela evaluated providers’ quality in a different manner in the previous and next procurements in 2006 and 2014. Thus, concentrating on choices and switches from one contract period simplifies the empirical analysis.

### Patient data

Our patient data is based on rehabilitation applications and invoices from the years 2010–2014. The applications data contains the patients’ age, sex, municipality, postcode and ICD-10 codes of primary, secondary and tertiary illnesses. The data also includes information on whether the patient had the right to receive the service at home, the number of annual physiotherapy sessions, the length of the sessions in minutes, and the number of years the patient had received the service since 2000. We have merged these applications with invoices based on the patient’s encrypted social security number. Providers were instructed to invoice Kela once a month, and therefore the data includes, on average, 10 annual observations for each patient. The data does not specify the date of each invoice or the number of visits each invoice holds, so we have calculated the annual number of invoices for each patient at every provider, and we consider the provider with the most invoices as the selected provider for each year.^10^ The invoice data also includes information on whether the patient received the service at home, based on whether the provider was paid extra for travelling. Unfortunately, we cannot observe the choice of a single physiotherapist or their flow between different providers.

Overall our data includes 17,963 patients and 64,252 different patient-provider observations in 2011–2014. We have excluded all patients who were under the age of 16 for three reasons: first, the eligibility criteria for the service are different for persons under the age of 16 than for those aged 16–65. Second, in empirical studies it is always very difficult to determine who makes the actual decisions (Beckert et al. 2012). However, it is likely that parents make choices for their children. Finally, this also improves the precision of the analysis because children are more likely to receive the service at school or kindergarten rather than at provider or home. We have also excluded all patients who either lived or visited a provider in an insurance district that implemented fixed prices during 2011–2014 as well as patients who travelled more than 100 km.

Our data enables us to identify new patients and those experienced with the service. We can also identify forced and active switchers based on patients’ previous choices. Active switchers decided to switch voluntarily, whereas forced switchers had to switch because their previous provider was not included in the pool of providers. Our main sample includes 2983 new patients, 555 patients who were forced to switch a total of 559 times, and 1679 active switchers who made 1,955 switches. Thus, in total we examine 5497 choices made by 5217 patients.

### Provider data

Our provider data includes all providers that met the minimum criteria in the 2010 competitive bidding (N = 1325). The data includes providers’ quality scores, their price for a 45-min physiotherapy session, reported annual capacity, address and information on whether the provider had the premises to provide the service.

Quality is multidimensional in health services and different quality measures can be based on inputs or outcomes (Tay 2003). Besides the providers’ overall quality score, the main quality attributes in this study are providers’ investments in their experience, education, facilities and equipment. These type of quality inputs can be considered as a proxy for providers’ underlying performance quality or utility gain construct (Forder and Allan 2014). Patients also listed these issues among the most important factors for their choice of provider in a survey (Pitkänen and Pekola 2016). Many of these quality aspects, such as physiotherapists’ additional training and professional experience, are also visible to patients if they compare alternative providers for example on the internet. Unfortunately, Kela does not collect comparable data on the individuals’ rehabilitation outcomes.

All of the quality categories were evaluated and scored in a similar manner in each insurance district, expect in the two districts that piloted fixed prices for the contract period 2011–2014. We have excluded all 123 providers from these two districts from the data, as well as seven providers who were rejected based on their quality-price ratio and 21 providers that did not sign the contract. Our final pool of providers includes 1174 providers. There were 67 providers that ended their contract during the contract period. We have excluded these providers from the years that followed their market exit, and the annual data on available providers includes only providers with a written contract. We have also calculated providers’ annual observed volume and the free capacity. The free capacity at a given year is calculated based on providers’ observed volume at previous year.

Table 1 describes the data on providers in the pool after the 2010 procurement. Providers scored an average of 80.5 points for total quality. The lowest quality score was 31 points and the highest score 104, the maximum score being 105. Providers’ average price was 47.5 euros, ranging from 28 to 99 euros. Because providers were accepted based on their quality-price ratio, the quality scores are correlated with prices (*r* = 0.37). However, the prices do not fully represent competitive prices, because the procurement mechanism was rather inefficient as only very few providers were not offered a contract (Pitkänen et al. 2020).

**Table 1:** Descriptive statistics of the providers

Variable	Obs	Mean	SD	Min	Max
*Quality scores*					
Education	1.174	13.43	6.08	0	20
Experience	1.174	21.54	6.68	0	30
Facilities	1.174	3.63	1.67	0	6
Equipment	1.174	5.41	1.32	0	6
Standard	1.174	35.96	4.84	0	41
Language skills	1.174	0.57	0.72	0	2
Total	1.174	80.54	13.63	31	104
*Characteristics*					
Price	1.174	47.48	7.59	28	99
Observed volume	1.174	10.92	14.59	0	153
Capacity	1.174	33.89	43.48	1	420
Free capacity	1.174	23.64	34.39	– 46	301
Premises	1.174	0.92	0.27	0	1
New	1.174	0.15	0.35	0	1

Table 1 also shows that on average, providers had an annual capacity for around 34 patients and an observed volume of nearly 11 patients in 2010. Thus, providers’ average free capacity at the beginning of the contract period was nearly 24. The total annual capacity of the accepted providers was around 39,800, which was almost three times the number of patients who received the service. The data also shows that some providers had more patients than their reported capacity. On the other hand, the reported capacity did not bind providers to take that number of patients. Providers were also not guaranteed to receive any patients, because patients were freely able to select their provider. Finally, 92% of the providers had their own facilities and 15% were new service providers.

### Choice sets

Patients can choose their provider from all accepted providersduring the contract period. Thus, we have merged our 2010 provider data with the patients in 2011–2014. We have calculated straight-line distances between each patient and all providers using the centre points of postcodes with an open data source from Statistics Finland.^11^ In order to create realistic choice sets based on the institutional features and to ease the computational burden, the choice sets include all providers in the patient’s own insurance district and all other providers within 80 km.^12^

## Methods

### Choice model

We use a random utility choice model by McFadden (1974). We assume that patients are rational and maximise their utility when choosing a provider. Patients weigh the distance against the providers’ quality and available times, measured as providers’ free capacity. Thus, the utility for a patient *i* at provider *j* at time *t* is:

1 $$U_{ijt} = V_{ijt} + e_{ijt} = \beta_{q} Q_{jt} + \beta_{c} C_{jt - 1} + \beta_{d} D_{ij} + \beta_{{d^{2} }} D^{2}_{ij} + e_{ijt} ,$$

where ${V}_{ijt}$ represents the observable utility, which depends on the provider’s quality ${Q}_{jt}$, free capacity ${C}_{jt-1}$ and distance ${D}_{ij}$ to alternative providers. However, we expect that the demand for the service is inelastic with respect to distance for patients who receive the service at their home. The error term ${e}_{ijt}$ includes unobserved provider characteristics, random utility, and the difference between perceived and true quality that is caused by asymmetric information. We assume that the patient’s utility from a provider is based on its quality in the 2010 competitive bidding, because all providers adhered to maintain their quality at the level of their tender for the entire contract period.

Patients choose from a set of alternative providers ${N}_{jt}$. Provider *j* is chosen if it results in the highest utility in the choice set. This indicates that active switchers decided to switch because their new provider resulted in a higher utility than their previous provider. We assume that the error term ${e}_{ijt}$ is independently and identically distributed (IID) with a type-1 extreme value distribution. This leads to a conditional logit model where the probability that a patient *i* selects provider *j* is:

2 $$Pr_{ijt} = \frac{{{\text{exp}}\left( {V_{ijt} } \right)}}{{\mathop \sum \nolimits_{{j^{\prime} \in M_{it} }} {\text{exp}}\left( {V_{{ij^{\prime}t}} } \right)}}.$$

### Methods

We estimate discrete choice models where the dependent variable is a dummy variable that receives a value 1 when patient *i* has chosen provider *j* and 0 for all other providers in the patient’s choice set. The alternative-specific variables in the choice sets are distance to each provider, their quality measures and free capacity. We estimate conditional logit models specified in (1) separately for new patients, forced switchers and active switchers. We allow a non-linear effect of distance on utility in all models by including linear and squared terms. The estimated coefficients are marginal utilities. All of our choice models are estimated using Stata 13 with the command *clogit*.

The estimated marginal utilities only provide information about the sign of the utility, whereas the ratio of marginal utilities provides quantitative information on patient’s preferences (Gutacker et al. 2016). These ratios of marginal utilities are also invariant with respect to the scale of the utility, and therefore a simple comparison of the ratios for different patient samples also gives us valuable information about the differences in their preferences (Santos et al. 2017). Thus, following this previous literature we estimate the patients’ willingness to travel (WTT) as:

3 $$WTT = \frac{{ - \beta_{q} }}{{\beta_{d} + 2\beta_{{d^{2} }} \overline{d}}},$$

where $\stackrel{-}{d}$ is the average distance to the chosen provider. WTT is the number of extra kilometres that a patient located at the average distance from a provider would be willing to travel if the provider’s quality measure increased by one point (Santos et al. 2017). We follow Hole (2007) and use the delta method (*nlcom*) to calculate the standard errors for the WTT estimates.

### Endogeneity

To interpret ${\beta }_{q}$ as a causal relationship, the error term should be uncorrelated with the independent variables. Previous literature has pointed four potential reasons why this might not hold in patient choice models (see Gutacker et al. 2016). First, providers with higher demand are more likely to make greater investments in their quality, which induces reverse causation. This concern is similar to the hospital choice models, where hospitals might learn by doing so that higher volume providers have higher quality (Gaynor et al. 2005). In this study, the provider quality data comes from the 2010 procurement, whereas all choices occurred in 2011–2014. Intuitively, these choices in the contract period cannot affect providers’ quality in the past procurement, but the future. Thus, we believe that the simultaneity arising from the effect of demand on quality is not a problem with our data and modelling approach.

Second, because providers have capacity constraints, high-quality providers might have less free capacity if demand is responsive to quality. This concern is similar to the endogeneity of waiting times in models for hospital demand (Gaynor et al. 2016). Again, we tackle this concern using lagged measure of free capacity that based on providers’ observed volume in the previous year. This approach of using lagged measures of both quality and capacity is similar to the previous studies (Gutacker et al. 2016; Santos et al. 2017; Varkevisser et al. 2012).

Third potential reason for endogeneity arises if there is systematic selection of patients that is not controlled for in the providers’ observed quality (Gutacker et al. 2016). However, the quality aspects used in our study reflect providers’ long-term quality investments and do not measure outcomes that patients’ choices or characteristics would have a direct influence. Thus, we believe that even if such systematic selection would occur, it does not influence the quality scores used in our study.

Fourth, unobserved provider characteristics that affect patient’s choices and provider quality may also contribute to endogeneity (Gutacker et al. 2016; Santos et al. 2017). Some of the previous studies have used a control group of urgent patients who should be less responsive to quality differences (Gutacker et al. 2016; Pope 2009). Unfortunately, our setting does not have such a control group, even though many forced switchers had only little time to choose their provider. The relationship between quality and demand may be of interest even without a strict causal interpretation, as it shows whether patients favor high-quality providers (Gutacker et al. 2016). In this study, we focus on the differences between experienced and new patients, and between those who receive the service at home and those who travel. Thus, our findings may reveal some important aspects of patient behavior in health care services.

## Results

### Descriptive evidence

Table 2 shows the descriptive statistics of new patients, forced switchers and active switchers. New patients are on average 50 years old, 54% of them are male and they receive 58 sessions in a year. Forced switchers are on average 46 years old and 50 percent of them are male. They receive 64 sessions annually and have received the service for nearly 9 years since 2000. Active switchers are the youngest group, the average age being 41, and 46 percent of them are male. They receive 62 sessions in a year and have received the service for 9 years since 2000. Over 50 percent of the patients in all these groups had the right to receive the service at home, but less than 40 percent actually received the service at home.

**Table 2:** Descriptive statistics of new patients, forced switchers and active switchers

Variable	New patients					Forced switchers					Active switchers				
	Obs	Mean	SD	Min	Max	Obs	Mean	SD	Min	Max	Obs	Mean	SD	Min	Max
*Patient characteristics*															
Age	2.983	50.20	12.58	16	65	559	45.81	14.47	16	65	1.955	40.52	16.08	16	66
Male	2.983	0.54	0.50	0	1	559	0.50	0.50	0	1	1.955	0.46	0.50	0	1
Right to sessions at home	2.983	0.50	0.50	0	1	559	0.54	0.50	0	1	1.955	0.56	0.50	0	1
Received sessions at home	2.983	0.35	0.48	0	1	559	0.38	0.49	0	1	1.955	0.38	0.49	0	1
Number of annual sessions	2.983	57.69	27.53	2	150	559	64.05	25.14	8	150	1.955	62.09	26.44	7	150
Length of a session (in min)	2.983	57.84	7.19	45	90	559	58.98	8.46	45	120	1.955	59.36	7.55	45	120
Years of rehabilitation	2.983	0	0	0	0	559	8.55	3.49	1	14	1.955	8.97	4.16	1	14
Number of illnesses	2.983	1.80	0.84	1	3	559	1.68	0.81	1	3	1.955	1.76	0.84	1	3
*Choice characteristics*															
Provider's quality	2.983	86.89	10.69	31	104	559	86.19	11.74	44	104	1.955	86.77	11.35	31	104
Previous quality	–	–	–	–	–	–	–	–	–	–	1.603	86.28	11.38	43	104
Distance to provider (in km)	2.983	10.31	13.24	0.5	99.51	559	11.81	12.33	0.5	99.11	1.955	13.29	15.97	0.5	99.59
Previous distance (in km)	–	–	–	–	–	559	10.25	12.09	0.5	95.11	1.955	15.08	17.96	0.5	99.51
Provider in same district	2.983	0.93	0.26	0	1	559	0.86	0.35	0	1	1.955	0.84	0.36	0	1
Distance to closest provider	2.983	4.10	7.59	0.5	99.11	559	4.69	8.16	0.5	95.11	1.955	3.40	6.42	0.5	62.80
Providers in 10 km radius	2.983	15.24	21.74	0	105	559	23.15	30.20	0	105	1.955	19.76	24.63	0	105
Providers in same district	2.983	57.41	16.53	4	84	559	58.80	17.68	4	84	1.955	57.49	16.02	4	84
Providers in choice set	2.983	127.10	80.70	4	327	559	155.78	88.68	4	330	1.955	142.67	86.43	4	328
Number of patients	2.983					555					1.679				

Patients in all groups have on average more than 15 providers within a 10 km radius and over 125 providers in their choice sets. On average, patients in all three groups choose a provider with higher quality than the average quality (80.5) of the accepted providers. However, there is no significant difference between the selected provider’s average quality among the three patient groups. We also compared the quality of the new and previous provider regarding 1,603 active switches that were made in 2012–14. On average, active switchers choose equally high quality providers as their previous ones. Thus, this evidence does not indicate that active switchers would systematically aim for a higher quality provider.

Table 2 also shows that the average distance from home to the chosen provider is 10.3 km for new patients, 11.8 km for forced switchers and 13.6 km for active switchers. Forced and active switchers are also more likely to choose the provider from another insurance district than new patients. Forced switchers choose a provider located 1.5 km further than their previous provider, whereas active switchers choose a provider 1.9 km closer. A potential explanation could be that many forced switchers were forced to travel further than previously, whereas many active switchers decided to switch to a closer provider.

**Fig. 1:**
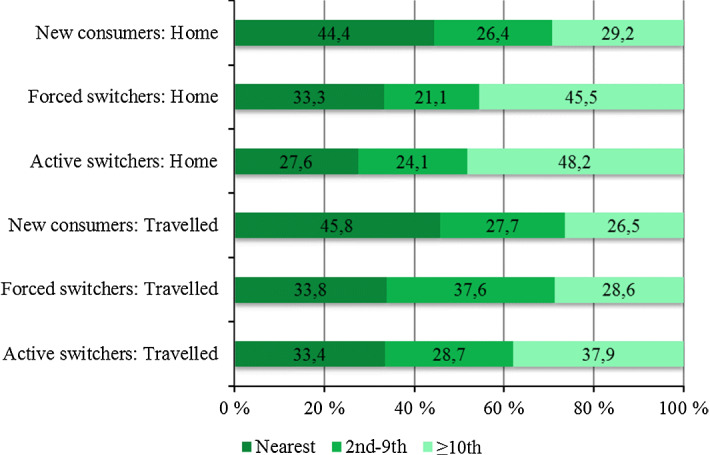
Percentage of patients who chose their nth nearest provider

Figure 1 shows that a majority of all patients bypass the nearest provider: 46 percent of new patients who need to travel choose their nearest provider, whereas only 34 percent of forced switchers and 33 percent of active switchers choose their nearest alternative. Our data also reveals that patients who receive the service at home select a provider further (12.1 km) than patients who need to travel (11.2 km), and they are also more likely to bypass the nearest provider regardless of their previous experience.

### Regression results

Table 3 presents our baseline estimation results from the conditional logit model of choice specified in Eq. (1) for new patients, forced switchers and active switchers during the 2011–2014 contract period. Quality of the provider is measured as the total quality score of each provider that is a lagged value from 2010. The main effects also include distance measured in kilometres between the postcodes of the patient and provider, a squared term of the distance and the free capacity for each provider that is based on previous year’s patient volume. Table 3 shows that, in general, all patients are more likely to choose high-quality physiotherapy providers and prefer short distances. Patients are also more likely to choose providers with greater free capacity. The estimated WTT for a one point increase in the total quality score is little over 0.5 kms for both new patients and active switchers. However, there is no similar statistically significant effect regarding the WTT for quality among forced switchers.

**Table 3:** Estimated marginal utilities: conditional logit models

Variable	New patients		Forced switchers		Active switchers	
	Est	SE	Est	SE	Est	SE
*Main effects*						
Quality	0.074	0.011***	0.051	0.022*	0.043	0.011***
Distance	− 0.136	0.013***	− 0.091	0.042*	− 0.085	0.011***
Distance^2^	0.0003	0.0001***	− 0.001	0.001	0.0001	0.0001
Free capacity	0.006	0.002***	0.011	0.003**	0.009	0.002***
*Interaction with quality*						
x Age	− 0.001	0.0002**	− 0.0004	0.0003	− 0.0003	0.0001*
x Male	− 0.010	0.004*	0.006	0.009	− 0.002	0.005
x Number of annual sessions	0.0001	0.0001	− 0.0005	0.0002**	0.0001	0.0001
x Number of illnesses	0.002	0.002	0.013	0.006*	− 0.0002	0.003
x Sessions at home	− 0.020	0.004***	0.008	0.009	0.003	0.005
*Interaction with distance*						
x Age	− 0.001	0.0002***	− 0.001	0.001	− 0.001	0.0002***
x Male	0.015	0.005**	− 0.038	0.020	− 0.012	0.005*
x Number of annual sessions	0.0001	0.0001	0.0001	0.0003	0.0001	0.0001
x Number of illnesses	0.001	0.003	0.014	0.012	0.0003	0.004
x Sessions at home	0.015	0.005**	− 0.004	0.021	0.020	0.005***
*Interaction with free capacity*						
x Age	0.0001	0.0001	− 0.0001	0.0001	0.0001	0.0002
x Male	0.0001	0.0001	− 0.001	0.001	0.0001	0.0001
x Number of annual sessions	0.0001	0.0001	0.0001	0.0001	− 0.0001	0.0001
x Number of illnesses	0.0001	0.0003	− 0.001	0.001	− 0.001	0.001
x Sessions at home	− 0.0007	0.0007	− 0.0001	0.001	− 0.002	0.001*
WTT (quality)	0.549	0.090***	0.546	0.335	0.513	0.139***
Number of patients	2,983		551		1,679	
Number of choices	2,983		555		1,955	
Number of observations	379,141		87,082		278,914	
BIC	16,253		3,812		13,493	
Pseudo R^2^	0.425		0.346		0.293	

Conditional logit models of choice of physiotherapy provider for patients’ choices in years 2011–2014. Estimated coefficients are marginal utilities. Interactions on patient characteristics with distance^2^ are not reported (available from the authors). WTT is the coefficient on quality divided by the marginal utility on distance (${\beta }_{d}+ {2\beta }_{{d}^{2}})$ evaluated at the average distance to the chosen provider. ***p < 0.001; **p < 0.01; *p < 0.05

We also estimate a choice model with four separate quality measures that are the providers’ quality scores for experience, education, facilities and equipment. Table 4 presents the results of these regressions. We find that new patients prefer providers with better education and experience. These results suggest that new patients search for providers already experienced in treating many disabled individuals for a long time. The results show that active switchers prefer providers with better experience and equipment. We do not find any statistically significant specific preferences for forced switchers. The results show that new patients are willing to travel 0.5 km for a one-point increase in the education score and 0.9 km for an increase in experience. Active switchers are also willing to travel 0.6 km for a one-point increase in experience and 6.3 km for an increase in the equipment score. Potential explanations for these results are that active switchers have learned about certain important quality features whereas forced switchers might have had only limited time to choose their new provider and have not thoroughly compared different alternatives.

**Table 4:** Estimated marginal utilities: conditional logit models

	New patients		Forced switchers		Active switchers	
Variable	Est	SE	Est	SE	Est	SE
Main effects						
Education	0.065	0.022**	0.034	0.047	0.011	0.023
Experience	0.124	0.022***	0.074	0.044	0.050	0.022*
Facilities	− 0.091	0.075	− 0.113	0.175	0.042	0.081
Equipment	0.275	0.147	0.799	0.431	0.535	0.165**
Distance	− 0.136	0.013***	− 0.010	0.042*	− 0.084	0.011***
Distance^2^	0.0003	0.0001***	− 0.001	0.001	0.0001	0.0001
Free capacity	0.007	0.002***	0.011	0.003**	0.009	0.002***
*Interaction with education*						
x Age	− 0.001	0.0003**	− 0.001	0.001	0.0001	0.0003
x Male	− 0.012	0.009	− 0.014	0.018	− 0.032	0.010***
x Number of annual sessions	0.0001	0.0001	− 0.001	0.0004	− 0.0001	0.0001
x Number of illnesses	0.003	0.005	0.034	0.012**	0.002	0.006
x Sessions at home	− 0.014	0.009	0.008	0.019	0.005	0.010
*Interaction with experience*						
x Age	− 0.0005	0.0003	− 0.001	0.001	− 0.001	0.0003**
x Male	− 0.007	0.009	0.025	0.018	0.025	0.010**
x Number of annual sessions	− 0.0002	0.0002	− 0.0004	0.0004	0.0003	0.0002
x Number of illnesses	− 0.003	0.005	0.006	0.012	0.003	0.006
x Sessions at home	− 0.013	0.009	0.0002	0.019	0.020	0.010
*Interaction with facilities*						
x Age	0.004	0.001**	0.002	0.002	0.002	0.001
x Male	0.017	0.031	0.031	0.070	0.054	0.035
x Number of annual sessions	0.001	0.001	0.001	0.001	0.001	0.001
x Number of illnesses	− 0.017	0.018	0.008	0.044	− 0.035	0.021
x Sessions at home	− 0.112	0.031***	− 0.035	0.073	− 0.178	0.036***
*Interaction with equipment*						
x Age	0.0005	0.002	0.002	0.006	− 0.005	0.002*
x Male	− 0.054	0.058	0.386	0.193*	− 0.055	0.071
x Number of annual sessions	− 0.0001	0.001	− 0.007	0.003*	0.0002	0.001
x Number of illnesses	0.034	0.034	− 0.120	0.100	− 0.006	0.043
x Sessions at home	− 0.303	0.060***	− 0.025	0.170	− 0.197	0.073***
*Interaction with distance*						
x Age	− 0.001	0.0001***	− 0.001	0.001	− 0.001	0.0002***
x Male	0.015	0.005**	− 0.041	0.020*	− 0.012	0.005*
x Number of annual sessions	0.0002	0.0001*	0.0001	0.0003	0.0001	0.0001
x Number of illnesses	0.001	0.003	0.016	0.012	0.0003	0.004
x Sessions at home	0.015	0.005**	− 0.005	0.021	0.020	0.005***
*Interaction with free capacity*						
x Age	0.0001	0.0001	− 0.0001	0.0001	0.0001	0.0001
x Male	− 0.0001	0.0001	− 0.001	0.001	− 0.0001	0.001
x Number of annual sessions	0.0001	0.0001	0.0001	0.0001	− 0.0001	0.001
x Number of illnesses	0.0001	0.0001	− 0.0002	0.001	− 0.001	0.001
x Sessions at home	− 0.0001	0.001	0.0004	0.002	− 0.001	0.001
WTT (education)	0.487	0.163**	0.322	0.485	0.131	0.269
WTT (experience)	0.917	0.182***	0.725	0.531	0.593	0.265*
WTT (facilities)	− 0.663	0.558	− 1.353	1.862	0.496	0.960
WTT (equipment)	2.028	1.096	8.003	5.485	6.345	2.095**
Number of patients	2,983		551		1,679	
Number of choices	2,983		555		1,955	
Number of observations	379,141		87,082		278,914	
BIC	16,290		3,957		13,575	
Pseudo R^2^	0.432		0.357		0.300	

Conditional logit models of choice of physiotherapy provider for patients’ choices in years 2011–2014. Estimated coefficients are marginal utilities. Interactions on patient characteristics with distance^2^ are not reported (available from the authors). WTT is the coefficient on quality measure divided by the marginal utility on distance (${\beta }_{d}+ {2\beta }_{{d}^{2}})$ evaluated at the average distance to the chosen provider. *** p < 0.001; ** p < 0.01; * p < 0.05

We examine potential patient heterogeneity that is captured through the interaction terms in the models. Like previous studies (Beckert et al. 2012; Beukers et al. 2014; Gutacker et al. 2016) we find that among new patients and active switchers, older individuals prefer shorter distances. We find no systematic difference between the preferences of female and male patients or patients who receive more annual sessions or have more illnesses. We find that those among new patients and active switchers who receive the sessions at their home are less likely to choose providers in close distance with high-quality facilities and equipment. These results are rather intuitive, as these patients are indifferent towards travel costs and time, and would not benefit from high-quality facilities.

## Conclusion

We are the first to study patients’ choices and switches of providers in a health service that is received frequently for a long time. Similar to previous studies, we find that patients prefer high-quality providers within short distances. Patients are also more likely to choose providers with greater free capacity. In addition, the results show that the willingness to travel for quality is highest among new patients and lowest among forced switchers. The results show that new patients especially prefer providers with better quality scores in education and experience. This indicates that new patients search for very experienced providers. Active switchers choose providers with high experience and equipment scores, whereas forced switchers seem to prefer experience and equipment. However, we find that especially new patients and active switchers are willing to travel for an increase in quality scores, whereas the effect is not statistically significant for forced switchers.

A potential explanation for our results is that new patients and active switchers have compared different providers more thoroughly than forced switchers. Active switchers may have learned about certain important quality aspects, as some of them have received the service for many years. However, our descriptive evidence does not indicate that patients who actively switch their provider systematically aim for a higher quality provider. Meanwhile, forced switchers may have had only limited time to compare different alternatives and choose their new provider. We also find a very intuitive result that patients who travel prefer shorter distances and high-quality facilities compared to patients who receive the sessions at their home.

Our findings show that patients are sensitive to quality differences among providers even when they are disabled and choose from a large set of alternative providers without quality information. The aim of the rehabilitation services is to promote the individuals’ autonomy and improve or maintain their work capacity and functioning. From a policy perspective it is important that patients are sensitive to quality and receive effective physiotherapy during the entire rehabilitation process. However, we find that forced switchers are not as responsive to quality as new patients or active switchers. Support for their choices through quality information or the use of separate contracts with previous providers could be useful. Overall, information about providers’ location and quality in an easily accessible form is a necessary condition for successful provider competition (Barros et al. 2016). Information would support all disabled individuals in their choice of physiotherapy provider and possibly encourage more patients to switch their provider.

Procurements for many health services are often organized in a repeated manner. This might cause challenges especially in services where patients receive the service frequently and have established a relationship with their usual provider. The repeated competitive biddings organized by Kela for this particular service were organized inefficiently, because nearly all providers were offered a contract (Pitkänen et al. 2020). This enabled choices from a large pool of providers and only a few patients needed to switch their usual provider. However, Kela changed its procurement practice in 2018, when nearly a third of the providers did not receive a contract. Thus, all of their usual patients were forced to switch to another provider. On the other hand, an efficient procurement ensures that the pool of providers consists of mainly high-quality providers, and that all patients receive the service cost-effectively.

Finally, our study has some limitations. We observe choices regarding providers and do not know the role and potential flow of individual physiotherapists working in these firms. Neither do we observe the actual decision-maker or the role of the physician as the referring agent, which is a common problem in empirical research (Beukers et al. 2014). Unfortunately we do not have quality information regarding individual rehabilitation outcomes. Also, the quality scores that we analyze were not publicly available for patients, and we do not have information regarding the providers’ specialization or their gender, which are also important factors behind choices. Finally, our study setting does not enable the use of a control group and the results should, therefore, be treated as relationships rather than causal effects.

## Footnotes

1 In 2010 there were 29 insurance districts that organized the competitive bidding but the number was reduced to 25 for the 2011–2014 contract period.

2 The maximum score was 20 for education, 30 for experience, 6 for facilities, 6 for equipment, 2 for language skills and 41 for Kela’s quality standard, which includes issues such as assessment of the rehabilitation process.

3 Free choice of providers was first mentioned in 2011 in a Kela service standard document. However, it was a common practice in medical rehabilitation services in the insurance districts already before 2011.

4 See https://asiointi.kela.fi/palvelutuottajarekisteri/ (Accessed: 30.3.2020).

5 86% of the providers had an informative website in December 2016.

6 This is a common issue also in other health and social services, because it is typical to organize the procurements repeatedly for example every four years.

7 The sessions can also take place for example at the patient’s school.

8 Payments for providers regarding home visits are calculated in the following way: For a travel distance under 36 km the payment is the minute-based price added by 50%, for a distance between 36 and 70 km added by 70%, and for a distance longer than 70 km added by 100%. Also, a separate travel cost is paid if the distance in one direction is more than 100 km. Thus, providers are compensated generously for their travel costs to patient’s home.

9 The reimbursement for patients’ travel costs has a co-payment for single one-way trips and an annual out-of-pocket maximum. Kela reimburses all costs that exceed the co-payment. The co-payments changed during the study period in the following way: In 2011–2012 the single trip co-payment was 9.25€ and the annual out-of-pocket maximum was 157.25€, whereas in 2013–2014 the single trip co-payment was 14.25€ and the annual out-of-pocket maximum was 242.25€. Travel costs exceeding the deductible must be reasonable, which in practice means that visits to providers in the same insurance district or within a close distance in other districts are covered. Travel costs are covered for the cheapest means of transport. For example, in 2015 Kela covered 88.4% of all patients’ travel expenses in medical rehabilitation and the most common means of transportation was taxi (63.7%).

10 Most patients commit themselves to one provider for a long time. However, some patients have single invoices from providers other than their permanent provider. According to Kela officials, most of these visits take place during vacation periods. In most cases the permanent provider has made this arrangement with another provider, and therefore we do not consider these visits patient choices.

11 We use a distance of 0.5 kms when the patient and provider were located in the same postcode. The smallest distance between two independent postcodes in the data was 0.93 kms.

12 An experienced Kela official recommended these choice sets.
